# Student Perceptions of Formative Clinical Feedback During Clerkships

**DOI:** 10.1007/s40670-026-02821-5

**Published:** 2026-07-20

**Authors:** Serena Schein, Isabel Banks, Paul Kunnath, Jasnoor Malhotra, Daniel T. Daly, Loki Cortner, Ashraf Jardaly, Kristina Dzara, Cynthia Nebel

**Affiliations:** 1 Department of Physical Medicine and Rehabilitation, Northwestern Feinberg School of Medicine, Chicago, IL USA; 2 Office of Curricular Affairs, Saint Louis University School of Medicine, Saint Louis, MO USA; 3 Department of Internal Medicine, Saint Louis University School of Medicine, Saint Louis, MO USA; 4 Department of Internal Medicine, Cleveland Clinic, Cleveland, OH USA; 5 Department of Surgery, Center for Anatomical Sciences and Education, Saint Louis University School of Medicine, Saint Louis, MO USA; 6 Department of Pediatrics, Wright Patterson Air Force Base, Fairborn, OH USA; 7 Department of Orthopedic Surgery, Saint Louis University School of Medicine, Saint Louis, MO USA; 8 Department of Family and Community Medicine, Saint Louis University School of Medicine, Saint Louis, MO USA; 9 Department of Psychiatry and Behavioral Neuroscience, Saint Louis University School of Medicine, Saint Louis, MO USA; 10 Center for Educator Development, Advancement, and Research, Saint Louis University School of Medicine, Saint Louis, MO USA; 11 Office of Student Affairs, Saint Louis University School of Medicine, 1402 S. Grand Boulevard, Caroline Hall 116, Saint Louis, MO USA

**Keywords:** Formative feedback, Medical education, Clerkship

## Abstract

The clerkship phase of medical school is essential to the development of clinical skills necessary for medical practice. This phase depends heavily on formative feedback to help students recognize and address their strengths and weaknesses. Recent student evaluation of formative clinical feedback at Saint Louis University revealed a potential barrier in student implementation of feedback due to negative perceptions of the accuracy and utility of the formative feedback they received. Using Norcini et al.’s Consensus Framework for Good Assessment, we developed a survey to investigate the medical student perception of our institution’s current system of formative feedback. Participants included 300 students in their third and fourth year medical students who had completed the clerkship phase. Response patterns indicated that, across years, Feasibility was rated highest and Catalytic Effect was rated lowest. Additionally, the only item rated higher than neutral was Feasibility, whereas Catalytic Effect, Equivalence, Acceptability, and Consistency were rated significantly below neutral. The patterns of results indicate (1) the practicality of the current formative feedback survey format and (2) students’ reduced perception of the usefulness of the formative clinical feedback they receive. While this student perception is subjective in nature, it is a key metric to consider as perception has the potential to drive behavioral change. Other medical schools can consider examining their process and student perception of formative feedback using a framework of assessment similar to the Norcini framework to promote continuous quality improvement in undergraduate medical education.

## Introduction

The undergraduate medical education clerkship phase is essential to the development of clinical skills necessary for residency and medical practice [1]. Throughout the clerkship phase, novice medical students rotate through various clinical settings which may include surgery, internal medicine, neurology, psychiatry, obstetrics and gynecology, pediatrics, and family medicine [2]. For many students, this is their first involvement in patient care and their first opportunity to develop competencies such as note writing, physical examination, patient communication, and teamwork.

A key component of successful progression through the clerkship phase is quality formative feedback, which allows students to recognize and address strengths and areas of improvement within their clinical skill set [3]. Given the variability in clerkship settings, learning opportunities, and clinical knowledge acquisition, it is critical for students to gauge their own level of competence and guide their personal learning needs via high-quality formative feedback [4]. The potential benefit of formative feedback on student clinical development is clear. However, there are many challenges associated with obtaining quality formative feedback related to both the structure of formative feedback implementation as well as faculty and student perception of a chosen formative feedback system. Some possible barriers to the implementation of formative feedback include the short timeframe in which faculty are expected to adequately evaluate students, the process of training faculty evaluators, and the standards students are evaluated against. It has been suggested that formative feedback should focus on sub-competencies or milestones, which may help identify student competency levels and developmental trajectories [5].

Additionally, given the importance of formative clinical feedback, several studies have evaluated other aspects of its implementation. Bray et al. (2021) successfully improved consistency between assessors’ ratings of students by implementing a faculty development session based on the Association of American Medical Colleges (AAMC) entrustable professional activities (EPA) [5]. Because there were clear standards combined with effective training, assessors were able to translate this system of evaluation into their supervision standards. Soukoulis et al. (2021) provide further support for the use of EPAs [6] finding that they are effective for ongoing data measurements that could be used in summative decisions. However, their study did not assess student perceptions and did not consider the utility of formative versus summative feedback.

Just as important as the implementation of formative feedback, and often overlooked, is the perception of formative feedback among faculty and students. If students perceive formative feedback as useful and constructive, they are more likely to use that information to make individual improvements [7]. Student perceptions of formative feedback seem to vary with how it is delivered [8]. For example, they may question its validity if viewed as one of many checklist requirements or a source of concern if documented in writing. Another concern from students is the frequent disconnect between student and faculty perceptions of formative feedback. While faculty often perceive themselves as providing frequent formative feedback, students are less likely to recognize that they are receiving formative feedback in informal settings [9]. These perceptions can detract from the likelihood of students using feedback for clinical improvement.

Given the potential pitfalls of the formative feedback process, it is necessary to establish evidence-based guidelines for effective formative feedback. By collecting and utilizing data to determine the quality of educational programs, institutions may be better able to inform future curricular directions. Broadly, evaluation, including that of formative feedback, should include the following: reaction or learner satisfaction, engagement, and relevance; learning or changes in knowledge, skills, attitudes, confidence, and commitment; change in learner behavior or the application of what was learned; and results or the achievement of outcomes and indicators of progress towards those outcomes [10].

Among the variety of methods to evaluate formative feedback effectiveness, the current study utilized Norcini et al.’s Consensus Framework for Good Assessment [11]. Norcini’s framework emphasizes the difference between formative and summative assessments defining formative assessment as low stakes, informal, opportunistic, and meant to stimulate learning as well as stating that it works best when it (1) is embedded in the instructional process and/or clinical workflow, (2) provides specific and actionable feedback, (3) is ongoing, and (4) is timely.” (1103) Additionally, Norcini’s framework for good assessment utilizes seven defined core components which have been well-supported by previous evidence. High quality assessment should be: coherent, continuous, comprehensive, feasible, purpose driven, transparent, and free from bias. Appendix A presents each component of the Norcini’s Framework and its operational definition (reproduced from Norcini et al. 2018). This framework provides a clear-cut, established method for evaluating clinical feedback.

The use of Norcini’s framework for good assessment in other studies provides support for its implementation in this study. The framework’s definition of systems of assessment and their use within progress testing and programmatic assessment were included in the Ottawa 2020 consensus statement for programmatic assessment [12]. Norcini’s emphasis on the importance of an integrative approach, rather than the traditional summative approach to assessment, is used to demonstrate the importance of programmatic assessment in the optimization of learning and learner progression in problem-based learning [13]. Additionally, the principles of Norcini’s framework have served as guidelines for feedback and assessment in studies on medical student exam scores [14], direct observation of procedural skills in radiology [15], and location or time-flexible versions of medical school progress testing [16]. Therefore, given both its adaptability and acceptance in medical education literature, we used Norcini’s seven principles of good systems of assessment to develop the survey for the current study.

The formative feedback process, like any component of medical education, is subject to student scrutiny and may contain pitfalls. At our institution specifically, there has been some discontent with the formative feedback process that warrants further evaluation. Formative feedback is collected using a Qualtrics survey around the halfway point during each clerkship. Most of the survey items are the same across clerkships — gathering a medical history, physical exam skills, patient presentations, generating a differential diagnosis — but specific clerkship skills such as suturing or the psychiatric mental status exam are only evaluated in their specialized rotation. The survey is completed by both the medical student and an attending physician to identify the student’s strengths and areas of improvement. It is then recommended that students set up a meeting with the attending physician to further discuss the items on the survey. It should be noted that these same items are later evaluated on the summative student evaluation at the end of the clerkship. Student evaluation of formative clinical feedback at Saint Louis University is obtained through traditional course surveys, a recent LCME self-study in preparation for an accreditation visit, and annual Independent Student Analyses (ISAs). These steps revealed a potential concern in student perceptions of the accuracy and usefulness of the formative feedback they received.

The reasons for this concern are unclear and may relate to the structure of the feedback process itself, in faculty implementation, or in student expectations. Without a comprehensive understanding of the challenges impacting the current formative feedback process, effective changes to this crucial component of medical education cannot be made. Additionally, much of the current literature does not address the significance of perceptions of formative feedback. Therefore, this project utilized a survey to investigate our institution’s current formative feedback system, incorporating a more comprehensive understanding of the effectiveness of the process by interrogating student perceptions.

## Materials and Methods

### Setting and Participants

Saint Louis University School of Medicine is a medium-sized, private, Jesuit midwestern university. Students in the clerkship phase, or the third and fourth years of undergraduate medical education, were surveyed. There were 179 eligible third-year and 154 eligible fourth-year students.

### Survey

A survey was developed to capture each of the components of Norcini’s framework described above. The survey included 17 Likert-scale items, each corresponding to aspects of Norcini’s framework (see Appendix B for full survey and Appendix C for scoring guide for corresponding items). In addition, three open-ended items were included to contextualize the primary results of the Likert-scale items. The survey was developed using the 7-step AMEE guide [17]. The research team conducted a review of literature relevant to formative feedback within medical education, explored the method of assessment at Saint Louis University School of Medicine, discussed findings as a team to generate survey items using Norcini’s framework as a guide for good assessment, conducted cognitive interviews, reviewed inconsistencies and revised confusing survey questions, and confirmed the survey was finalized prior to distribution.

Survey items were developed to represent the seven components of effective formative feedback described by Norcini. Because the instrument was designed to characterize perceptions across multiple framework domains rather than to establish a unidimensional scale, the number of items representing each component varied according to the breadth and complexity of the domain. Components represented by multiple items were analyzed by averaging across items (see Appendix C for the scoring guide), whereas single-item components were retained as standalone indicators. The survey was designed to provide an estimate of student perceptions related to each of the seven components of Norcini’s framework for effective formative feedback rather than to develop a psychometric instrument. Accordingly, internal consistency reliability for the instrument was not assessed.

### Procedure

The project was approved by the Saint Louis University Institutional Review Board (#34408). Survey data were collected using Qualtrics. Third-year students were surveyed at a class gathering at the end of clinical rotations in December 2024. Fourth-year students were surveyed during their class capstone course during their final semester in March 2025, a little over a year after they finished their core clinical rotations. The survey was announced to both classes in-person, and a QR code was provided to access the survey during their respective meeting times. Students were reminded that participation was voluntary and consent was attained at the beginning of the survey. All students were offered a small refreshment for completing the survey but were not asked for evidence of their completion in order to maintain anonymity.

### Data Analysis

Survey response rates were 81.6% and 89.5% respectively. These response rates allow our findings to generalize to our population of third-year and fourth-year medical students, respectively, with 95% confidence in our findings and less than a 5% margin of error (Table 1) [18]. Many students responded to the free response questions about most (*N* = 180) and least (*N* = 156) helpful formative feedback experiences. A total of 193 students provided an additional comment.

**Table 1:** Survey response rates by cohort

Cohort	Eligible Students	Survey Responses	Response Rate
Third Year	179	146	81.6%
Fourth Year	172	154	89.5%
Total	351	300	85.5%

Data were first examined to ensure quality and completion. Two participants from the third-year cohort and one participant from the fourth-year cohort had no variability in responses and were removed from the dataset. In addition, 5 participants in the third-year cohort who completed less than 80% of the survey were removed from the dataset, resulting in final numbers of respondents of 139 and 153 respectively.

The eleven items associated with variability were reverse coded (e.g., 5s changed to 1s, 4s changed to 2s, etc.) to indicate that higher variability indicated worse feedback, and all questions associated with a given component of Norcini’s framework were averaged to arrive at a score for each participant on each component (see Appendix C). For Consistency, two participants in the third-year cohort and one participant in the fourth-year cohort did not have enough data to calculate a score, so the number of respondents was reduced for that item. For Validity and Equivalence, three participants in the third-year cohort and two participants in the fourth-year cohort were missing one value in each component. This missing data was handled by using the average of the remaining items for these components.

Because the data were aggregated across items and the number of respondents was reasonably large, the Likert scale data was treated as interval-level data, which is argued to be appropriate by Carifio and Perla (2008) [19]. A mixed-design ANOVA with year (M3 vs. M4) as the between-subjects factor and the seven Norcini framework components as the within-subjects factor revealed a significant interaction between year and Norcini component, *F*(6, 282) = 8.72, *p* < .001, *η*_*p*_^*2*^ = 0.03. Subsequent results are therefore reported for each cohort of third-year and fourth-year medical students separately and between-cohort comparisons are discussed.

Open-ended questions were analyzed via conventional content analysis [20]. Responses were organized into coding categories derived directly from the text data by open-ended question. Once organized into coding categories, the data were summarized by coding category to enable the further contextualization of student formative feedback experiences.

## Results

**Fig. 1:**
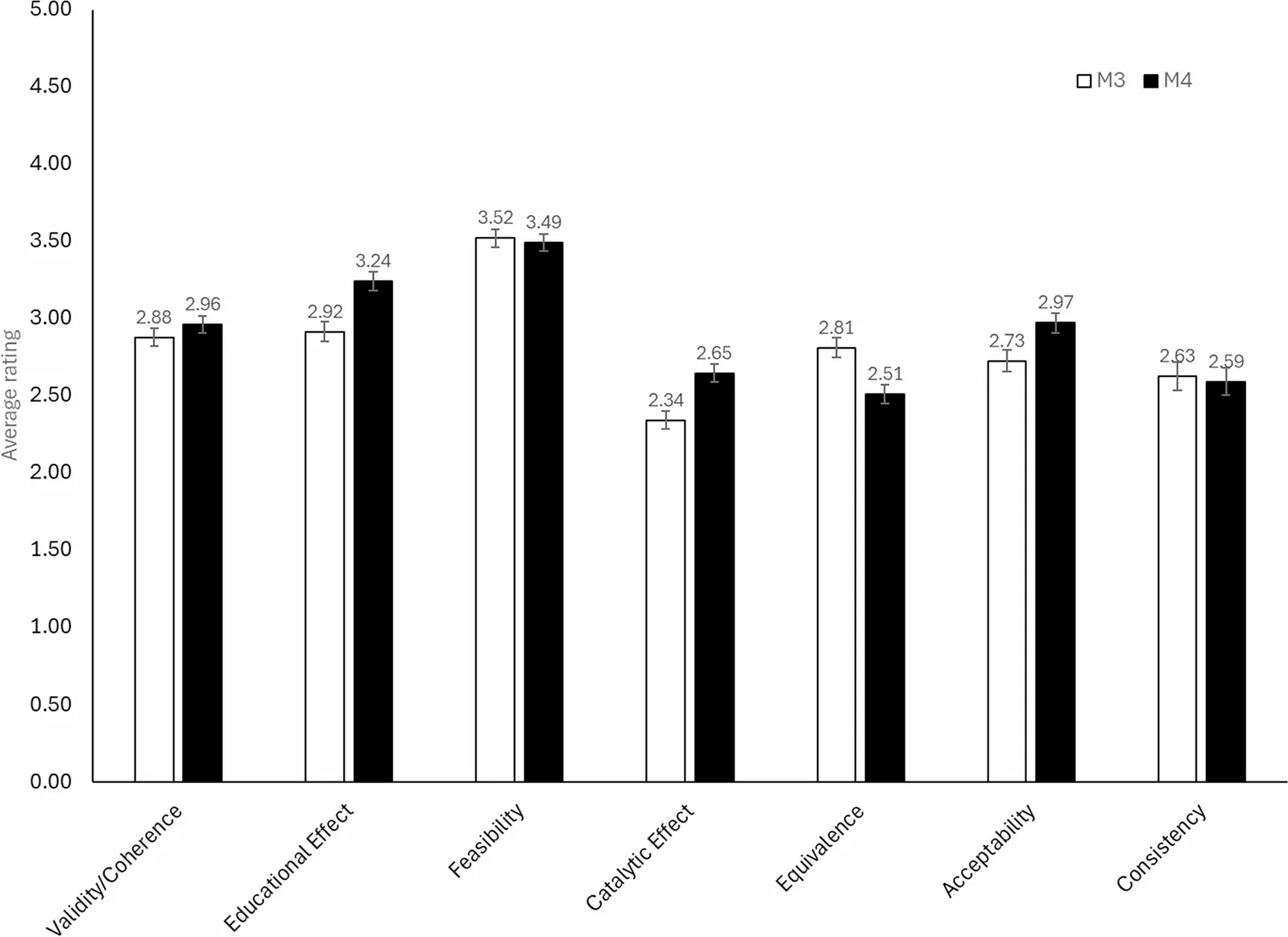
Means for each component of the Norcini framework for each cohort. Error bars represent standard error of the mean.

All means and standard deviations are displayed in Fig. 1. There was a significant main effect of Norcini component, *F* (6, 282) = 79.40, *p* < .001, *η*_*p*_^*2*^ = 0.22. Due to the large number of comparisons and the survey having been designed by the team for this study, a conservative approach was selected for comparisons between components. Follow-up paired t-tests were conducted using a Bonferroni correction to correct for multiple comparisons (α = 0.0025). Feasibility (*M* = 3.51) was rated significantly higher than all other components (all *p* < .001). Educational Effect (*M* = 3.08) was rated significantly higher than all remaining components (all *p* < .001). Catalytic Effect was rated significantly lower than all components (all *p* < .001) with the exception of Equivalence (*p* = .02) and Consistency (*p* = .10).

**Fig. 2:**
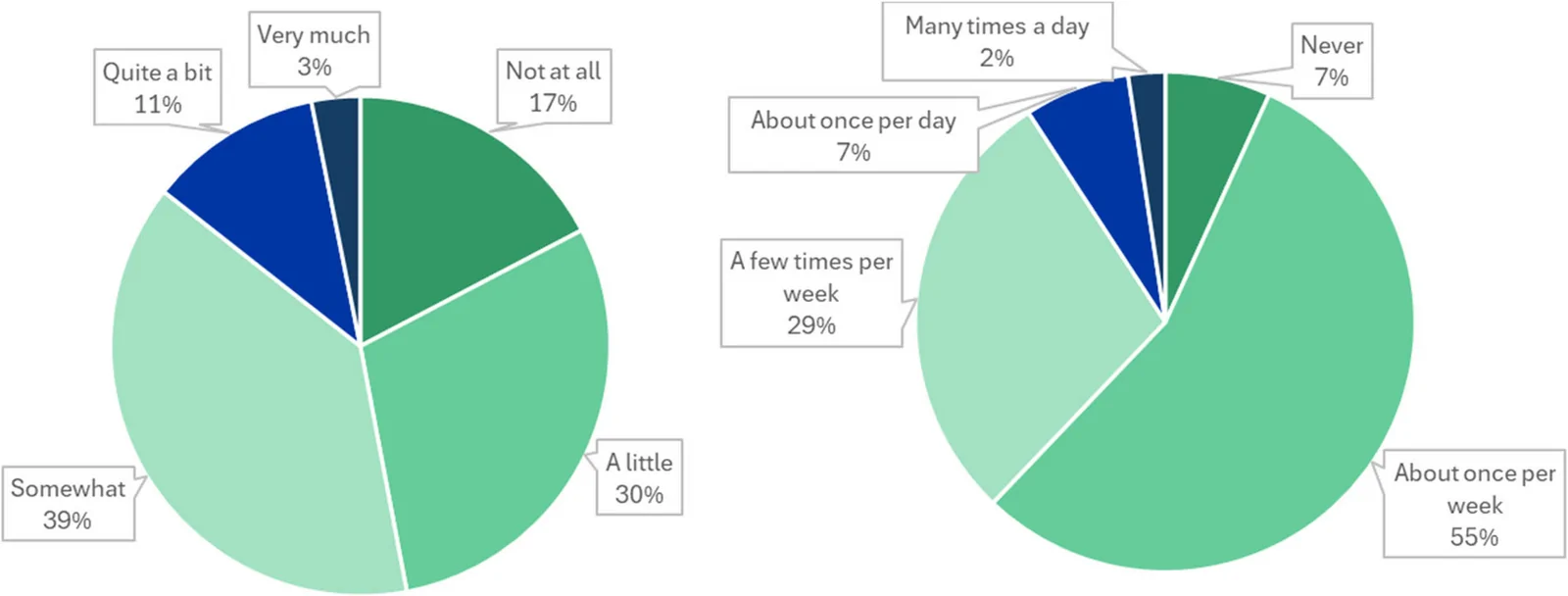
**a** and **b**. Response percentages across survey items addressing Catalytic Effect. Figure 2a displays the average proportion of responses across the three items with the same response options (items 5, 15, and 17). Figure 2b displays the proportion of responses for item 27

The lower mean for Catalytic Effect was concerning because arguably this is the component we would hope to be the highest for quality feedback – namely, that students perceive that they can use the feedback to make improvements. We therefore did additional analyses examining this component. There were four items that targeted Catalytic Effect, three of which used the same response scale and were therefore able to be combined. The number of responses for each answer choice were tallied and then averaged across the three items. We then calculated the average proportion of responses for each of the answer choices (see Fig. 2a). Our goal would be for students to perceive the catalytic effect of the formative feedback at a rating of quite a bit or very much, which combined only make up 14% of responses. Conversely, 17% of participants perceived that they were “not at all” able to use the formative feedback to improve their clinical performance. The final catalytic effect item asked about how often students perceived receiving informal formative feedback (Fig. 2b). Again, our ideal would be for students to perceive that they receive informal formative feedback on a daily basis, but only 9% of students perceived this to be true and 7% reported never receiving any informal formative feedback, with 55% of students perceiving that they only received informal formative feedback once per week.

### Third-year Cohort

As indicated above, while there was no main effect of cohort [*F* (6, 282) = 2.12, *p* = .15, *η*_*p*_^*2*^ = 0.007] the interaction between cohort and component was significant. For the third-year cohort, a repeated measures ANOVA on component indicated a significant main effect [*F* (6, 798) = 40.85, *p* < .001, *η*_*p*_^*2*^ = 0.24]. Follow-up paired t-tests were conducted using a Bonferroni correction to correct for multiple comparisons (α = 0.0025). Feasibility was rated as significantly higher than all other components (*p* < .001 for all comparisons) and Catalytic Effect was rated as significantly lower than all other components (*p* < .001 for all) with the exception of Consistency (*p* = .003).

As a secondary analysis, we compared the overall score on each component to a neutral rating of “3” on the Likert scale, again using a Bonferroni correction to account for multiple comparisons (α = 0.007). Feasibility was rated significantly higher than neutral, *t*(288) = 13.11, *p* < .001, *d* = 0.77. Catalytic Effect [*t*(288) = -12.10, *p* < .001, *d* = 0.71], Equivalence [*t*(288) = -8.03, *p* < .001, *d* = 0.47], Acceptability [*t*(288) = -2.86, *p* = .004, *d* = 0.17], and Consistency [*t*(288) = -6.22, *p* < .001, *d* = 0.37] were all rated significantly lower than neutral.

### Fourth- year Cohort

For the fourth-year cohort, a repeated measures ANOVA on component indicated a significant main effect, [*F* (9, 894) = 47.47, *p* < .001, *η*_*p*_^*2*^ = 0.24]. Follow-up paired t-tests were conducted using a Bonferroni correction to correct for multiple comparisons (α = 0.0025). All mean differences were significant with *p* < .001 with the following exceptions: Validity/Coherence and Acceptability (*p* = .71), Catalytic Effect and Equivalence (*p* = .09), Catalytic Effect and Consistency (*p* = .52), and Equivalence and Consistency (*p* = .41). Consistent with the third-year cohort, Feasibility was rated significantly higher than all components (all *p* < .001) and Catalytic Effect was rated significantly lower than all other components (all *p* < .001) with the exception of Equivalence (*p* = .09) and Consistency (*p* = .52).

As a secondary analysis, we compared the overall score on each component to a neutral rating of “3” on the Likert scale, again using a Bonferroni correction to account for multiple comparisons (α = 0.007). Feasibility [*t*(152) = 8.86, *p* < .001, *d* = 0.72] and Educational Effect [*t*(152) = 4.12, *p* < .001, *d* = 0.33] were rated significantly higher than neutral, Catalytic Effect [*t*(152) = -6.64, *p* < .001, *d* = 0.54], Equivalence [*t*(288) = -8.52, *p* < .001, *d* = 0.69], and Consistency [*t*(288) = -4.61, *p* < .001, *d* = 0.38] were all rated significantly lower than neutral.

### Additional Question Analysis

Means and standard deviations for each question can be found in Appendix B. The item with the lowest average rating (*M* = 2.43) asked “How often do you receive informal formative feedback outside of the mid-clerkship formative feedback evaluation?” followed by “To what extent are you able to use the mid-clerkship formative feedback to improve your clinical performance?” (*M* = 2.58).

The item with the highest average rating (*M* = 4.27) was “How easy is it to complete your part of the mid-clerkship formative feedback form?” followed by a rating (*M* = 4.07) that indicated little variability across clerkships as to whether students are observed enough (*M* = 2.72). All other ratings were less than *M* = 4.0.

### Open-ended Question Analysis

Open-ended questions asked respondents about their most and least helpful formative feedback experience to help contextualize our survey results. A small number of respondents reported that they always experience formative feedback as helpful. Numerous comments pointed to the importance of students sitting down and having a robust discussion with faculty evaluators to more adequately understand the feedback. Respondents also noted the importance of being adequately observed or having built a longitudinal relationship with faculty evaluators so that meaningful feedback can be provided and received. Actionable and specific feedback was valued and desired. Some chose to name specific departments in which they had positive experiences or specific faculty evaluators for whom they had a positive formative feedback experience.

Respondents found it least helpful when feedback was not actionable. They also noted having a negative feedback experience when the amount of faculty observation prior to receiving feedback was limited, or when they perceived minimal effort on the part of faculty evaluators who gave the same value for all categories on the formative feedback form without a clear rationale for doing so. In some cases, faculty evaluators did not ever give high scores but did not provide constructive feedback to give students an opportunity to increase their score. A number of respondents indicated that they submitted the formative feedback form and then did not meet with their faculty evaluator, or that they never received a copy of the information which was submitted. In some cases, feedback was simply not timely, and students did not have enough time to implement the formative feedback prior to the end of the rotation, especially for shorter rotations. Some chose to name specific departments in which they had a negative feedback experience.

Additional responses suggested that a small number of students find the experience to be a waste of time, attributed to variability by rotation and variability in faculty evaluator understanding of the formative feedback process. A few respondents indicated that residents were helpful to their learning process and should also engage in the formative feedback process.

## Discussion

300 students who had completed the clerkship phase (third- and fourth-year cohorts) completed our survey created using the Norcini et al. (2018) framework for good formative assessment [11]. Our primary findings indicate that, although the formative feedback process is relatively straightforward, not all students are using their formative feedback to make meaningful changes to their clincal performance. There was a significant difference between cohorts, which may represent a level of hindsight bias given that one cohort took the survey during their last clerkship block, and the other cohort took the survey a year after they had finished their clerkships.

Despite this difference, there was similarity in the response pattern across cohorts with Feasibility rated the highest, Catalytic Effect rated the lowest. With the goal of determining student perceptions of formative feedback and improving the process at our institution, student response data were analyzed compared to a neutral response. For example, when addressing catalytic effect, we hope students feel that they are using the formative feedback that they receive quite a bit or very much rather than somewhat, a little, or not at all. See Appendix B for other survey item Likert scales. Across cohorts, the only item rated higher than neutral was Feasibility, whereas Catalytic Effect, Equivalence, Acceptability, and Consistency were rated significantly below neutral. Free-text response data offered additional evidence in support of the findings above, by expanding on the variability across faculty evaluators and clerkships. Students offered some additional information about why formative feedback has low Catalytic Effect, namely that the feedback on the form is often not actionable and has limited utility when not followed up with an in-person discussion. The similar responses of both cohorts, demonstrated across several components of the Norcini framework, are indicative of (1) the practicality of the current formative feedback survey format and (2) the need to better understand student perceptions surrounding the reliability, credibility, and impact of formative clinical feedback.

The use of self-report surveys for data collection can introduce a level of subjectivity that makes interpretation more challenging. Although the research team used the 7-step AMEE guide [17] to generate clear, focused survey items, how each individual student interpreted the questions may be variable. Furthermore, the project team must interpret the reason for response patterns without participant reasoning. For a few survey items, the introduction of free response style questions helped elucidate some of these subjective interpretations when analyzing the data. Another issue with examining subjective data is determining whether it is accurate to reality. That is, these data do not tell us whether there is truly little catalytic effect of the formative feedback or whether students perceive little catalytic effect. However, the primary focus of this project was to evaluate student perceptions of formative feedback. If students do not recognize the catalytic value of the feedback they have received, they are unlikely to act on it, regardless of the true value.

Based on the 2026 AMEE guide for formative assessment and feedback in medical education, formative assessment should ideally take a “holistic approach that considers not only behavioral outcomes but also cognitive and emotional responses to feedback” [22]. Survey results inform us that student perceptions of the standardized formative feedback process at the Saint Louis University School of Medicine are variable and reveal possible limited utility. Student perceptions of the feedback are directly tied to their willingness to make changes, possibly explaining the lower ratings for many of the Catalytic Effect items.

Another notable finding is the higher student response rate in the “Never,” “About once per week,” and “A few times a week” categories compared to “About once per day” or “Many times a day” when asked about how often students are receiving informal formative feedback. Literature demonstrates that faculty perceive themselves as frequently providing formative feedback outside the scope of the formal assessment protocol during each rotation [23], but that students may not recognize that they are receiving formative feedback. This study did not evaluate faculty perceptions of formative feedback. However, the extant literature indicates that faculty value the functionality and usefulness of formative feedback in identifying student strengths and weaknesses and altering teaching plans to cover curricular gaps [21]. A possible solution to this issue may be that both students and faculty could receive training to better identify opportunities for informal formative feedback during the clerkship phase as well as how to make formative feedback actionable.

These concerns are not unique to our institution. Greater emphasis on formative feedback has been shown to improve subjective student motivation and engagement [24]. Other existing literature may suggest, however, that students have mixed feelings about formative feedback with concerns regarding the time it takes and the usefulness of the feedback itself [25]. Quality formative feedback should achieve a balance between feasibility and usefulness and become a dynamic process that supports student learning and growth rather than a checkbox or tool [22]. Questions relating to what other opportunities exist for more informal formative feedback and the context in which formative feedback is delivered remain. Like the existing literature, the disconnect demonstrated and the results revealing certain areas of dissatisfaction may represent areas where faculty and students can work together to facilitate learning and identify knowledge gaps in both a formal and an informal manner during the clerkship phase. Similar to a follow up study mentioned by Lim (2019), there may be benefits to conducting a future study collecting direct, subjective data from students in their own words. Lim notes that the degree of student involvement in the design and implementation of formative assessments also plays a role in its effectiveness [25]. This data could elucidate more of the reasoning and concerns underlying student dissatisfaction and the disconnect seen between faculty understanding of feedback importance and student perceptions of the process. To further explore the disconnect, it will be beneficial to examine faculty perceptions of the same formative feedback process.

At our institution, the collected data were used to generate a report to leadership highlighting the possible disconnect between formative feedback provided to students and their perceptions and willingness to utilize the recommendations in the clinical space. Based on these discussions, we implemented a new formative feedback process that is distinct from the summative assessment at the end of the clerkship, whereas the prior system assessed the same skills on both mid-clerkship and summative feedback forms. The new system involves a drop-down menu of EPA-based strengths and weaknesses. Faculty select three strengths and three weaknesses for each student they evaluate and must expand on their reasoning and thoughts in a free text box for one of each. With the new system, we aim to maintain feasibility and increase students’ understanding of why they are receiving the feedback and how they can improve. Although the system still leaves face-to-face meetings with preceptors an ongoing challenge, the more in-depth explanations may encourage students to ask questions and seek formative feedback on a daily basis.

## Conclusion

Quality formative feedback is crucial to the development of clinical skills during the clerkship phase of undergraduate medical education. However, some students have less than ideal perceptions of the current formative feedback process. This study helped to identify those perceptions as related to the usefulness of the feedback for students in making meaningful change at Saint Louis University School of Medicine. Given that medical student perceptions of formative feedback and their involvement in the system likely impact their willingness to implement changes in the clinical learning environment, it is necessary for institutions to explore this facet of the feedback process. By considering student perceptions of formative feedback and incorporating their thoughts into continuous quality improvement projects like this one, medical schools can enhance student learning. Other institutions can consider examining their own processes of formative feedback using a framework of assessment similar to the Norcini framework as well as incorporating faculty and student perceptions in their ongoing assessments to include a more holistic approach. By evaluating student perceptions, medical schools can consider the role of cognition and emotion in the feedback process to better address any concerns or pitfalls in their current system.

## Appendix A

**Table:**

Framework for Good Assessment: Systems of Assessment	
Validity or Coherence	The results of an assessment are appropriate for a particular purpose as demonstrated by a coherent body of evidence.
Reproducibility, Reliability, or Consistency	The results of the assessment would be the same if repeated under similar circumstances.
Equivalence	The same assessment yields equivalent scores or decisions when administered across different institutions or cycles of testing.
Feasibility	The assessment is practical, realistic, and sensible, given the circumstances and context.
Educational Effect	The assessment motivates those who take it to prepare in a fashion that has educational benefit.
Catalytic Effect	The assessment provides results and feedback in a fashion that motivates all stakeholders to create, enhance, and support education; it drives future learning forward and improves overall program quality.
Acceptability	Stakeholders find the assessment process and results to be credible.

### Appendix B

Student Survey Developed Based on Norcini et al.’s Consensus Framework for Good Assessment^11^.

You are being asked to participate in this survey because you are a student at the Saint Louis University School of Medicine. This survey is part of the PRIMED initiative at our institution to better understand student perceptions of formative evaluations of clinical performance. This has been approved by the Saint Louis University IRB.

Your participation is voluntary and there are no foreseeable risks. If at any time you feel uncomfortable, you can skip a question or stop taking the survey. The goal of this survey is to use what we learn to make recommendations to leadership about improving the formative evaluation process of clinical performance.

For the questions below, we are specifically asking about formative feedback. We define formative feedback as evaluation that occurs during a course, module, or experience that is meant to give a learner input about their performance prior to and excluding any summative assessments that determine their clerkship grade. For the questions that follow, consider what is most typical, even though there might be variability among different rotations.

If you have any questions about this survey, you can contact Dr. Cynthia Nebel, Director of Learning Services, at cynthia.nebel@health.slu.edu.

- (Validity/Coherence) How well are the questions on the mid clerkship formative evaluation written to accurately measure clinical performance? (*M =* 2.96, *SD =* 0.92)
- Response Options: Not at all, Slightly, Moderately, Pretty well, Very well.
- (Educational Effect) How accurately are you able to judge your own clinical performance? (*M* = 3.33, *SD* = 0.81)
- Response Options: Not at all, Slightly, Moderately, Pretty accurately, Very accurately.
- (Feasibility) How easy is it to complete your part of the mid-clerkship formative feedback form? (*M* = 4.27, *SD* = 81)
- Response Options: Very difficult, Difficult, Neutral, Easy, Very Easy.
- (Educational Effect) How much do you prioritize improving on the rating-scale criteria in the mid clerkship formative evaluation? (*M* = 3.06, *SD* = 1.20)
- Response Options: Not at all, A little, Somewhat, Quite a bit, Very much.
- (Catalytic Effect) To what extent are you able to use the mid-clerkship formative feedback to improve your clinical performance? (*M* = 2.58, *SD* = 0.96)
- Response Options: Not at all, A little, Somewhat, Quite a bit, Very much.
- (Educational Effect) How much does the mid-clerkship formative feedback motivate you to improve your clinical performance? (*M* = 2.85, *SD* = 1.06)
- Response Options: Not at all, A little, Somewhat, Quite a bit, Very much.
- (Validity/Coherence) Do the faculty conducting your mid-clerkship formative evaluation observe your clinical performance enough to make informed judgments? (*M* = 2.72, *SD* = 0.86)
- Response Options: Not at all, A little, Somewhat, Quite a bit, Very much.
- (Equivalence – Reverse coded) How much does this vary across clerkships? (*M* = 4.07, *SD* = 0.94)
- Response Options: Not at all, A little, Somewhat, Quite a bit, Very much.
- (Feasibility) How easy is it to select a mid-clerkship formative feedback evaluator? (*M* = 3.34, *SD* = 0.92)
- Response Options: Very difficult, Difficult, Neutral, Easy, Very Easy.
- (Equivalence – Reverse coded) How much does this vary across clerkships? (*M* = 3.43, *SD* = 1.17)
- Response Options: Not at all, A little, Somewhat, Quite a bit, Very much.
- (Feasibility) How easy is it to meet with a faculty evaluator to review the mid-clerkship formative feedback? (*M* = 2.92, *SD* = 0.92)
- Response Options: Very difficult, Difficult, Neutral, Easy, Very Easy.
- (Equivalence – Reverse coded) How much does this vary across clerkships? (*M* = 3.49, *SD* = 1.11)
- Response Options: Not at all, A little, Somewhat, Quite a bit, Very much.
- (Validity/Coherence) How well do the expectations of your clinical performance laid out for you during clerkship orientation match the criteria on the mid-clerkship formative evaluation? (*M* = 3.08, *SD* = 0.95)
- Response Options: Not at all, Slightly, Moderately, Pretty well, Very well.
- (Equivalence – Reverse coded) How much does this vary across clerkships? (*M* = 3.02, *SD* = 1.14)
- Response Options: Not at all, A little, Somewhat, Quite a bit, Very much.
- (Catalytic Effect) How useful is the mid-clerkship formative feedback for your understanding of your clinical performance? (*M* = 2.65, *SD* = 0.95)
- Response Options: Not at all, A little, Somewhat, Quite a bit, Very much.
- (Equivalence – Reverse coded) How much does this vary across clerkships? (*M* = 3.18, *SD* = 1.15)
- Response Options: Not at all, A little, Somewhat, Quite a bit, Very much.
- (Catalytic Effect) To what extent are improvements after your mid-clerkship formative feedback reflected in your summative assessment results? (*M* = 2.37, *SD* = 1.08)
- Response Options: Not at all, A little, Somewhat, Quite a bit, Very much.
- (Equivalence – Reverse coded) How much does this vary across clerkships? (*M* = 2.94, *SD* = 1.24)
- Response Options: Not at all, A little, Somewhat, Quite a bit, Very much.
- (Acceptability) How much trust do you have in the skills and abilities of your faculty evaluator to judge your mid-clerkship clinical performance? (*M* = 2.89, *SD* = 0.96)
- Response Options: Not at all, A little, Somewhat, Quite a bit, Very much.
- (Equivalence – Reverse coded) How much does this vary across clerkships? (*M* = 3.34, *SD* = 1.20)
- Response Options: Not at all, A little, Somewhat, Quite a bit, Very much.
- (Acceptability) How much does the mid-clerkship formative feedback received from faculty accurately reflect your clinical performance? (*M* = 2.82, *SD* = 0.88)
- Response Options: Not at all, A little, Somewhat, Quite a bit, Very much.
- (Equivalence – Reverse coded) How much does this vary across clerkships? (*M* = 3.32, *SD* = 1.11)
- Response Options: Not at all, A little, Somewhat, Quite a bit, Very much.
- (Consistency) How consistent are mid-clerkship ratings across different faculty evaluators when evaluating students with equivalent clinical performance? (*M* = 2.61, *SD* = 1.06)
- Response Options: Not at all, A little, Somewhat, Quite a bit, Very much.
- (Equivalence – Reverse coded) How much does this vary across clerkships? (*M* = 3.28, *SD* = 1.18)
- Response Options: Not at all, A little, Somewhat, Quite a bit, Very much.
- (Validity/Coherence) How consistently are criteria for clinical performance applied on the mid-clerkship and summative evaluations? (*M* = 2.89, *SD* = 0.93)
- Response Options: Not at all, A little, Somewhat, Quite a bit, Very much.
- (Equivalence – Reverse coded) How much does this vary across clerkships? (*M* = 3.06, *SD* = 1.12)
- Response Options: Not at all, A little, Somewhat, Quite a bit, Very much.
- (Catalytic Effect) How often do you receive informal formative feedback outside of the mid-clerkship formative feedback evaluation? (*M* = 2.43, *SD* = 0.82)
- Response Options: Never, About once per week, A few times a week, About once per day, Many times a day.
- (Equivalence – Reverse coded) How much does this vary across clerkships? (*M* = 3.68, *SD* = 1.16)
- Response Options: Not at all, A little, Somewhat, Quite a bit, Very much.

1. How many clerkship rotations have you completed? (number only)
2. Tell us your least helpful formative feedback experience.
3. Tell us your most helpful formative feedback experience.
4. Do you have any additional comments about the mid-clerkship formative feedback process that you would like to share with us?

### Appendix C

Instructions for scoring the survey:

1. Reverse code survey item numbers 8, 10, 12, 14, 16, 18, 20, 22, 24, 26, 28. (For example, change 5 to 1 and 4 to 2, etc.)
2. Calculate each participant’s average for each framework component:
  3. Validity/Coherence: survey item numbers 1, 7, 13, 25.
  4. Educational Effect: survey item numbers 2, 4, 6.
  5. Feasibility: survey item numbers 3, 9, 11.
  6. Catalytic Effect: survey item numbers 5, 15, 17, 27.
  7. Equivalence: survey item numbers 8, 10, 12, 14, 16, 18, 20, 22, 24, 26, 28.
  8. Acceptability: survey item numbers 19, 21.
  9. Consistency: survey item numbers 23.

## Declarations

### Prior Presentations

This work has previously been presented at the Saint Louis University School of Medicine AOA Research Forum, Science Day, and GME Housestaff Poster Competition, and at the University of Southern California Innovations in Medical Education Conference.
